# Distinguishing the Impacts of Inadequate Prey and Vessel Traffic on an Endangered Killer Whale (*Orcinus orca*) Population

**DOI:** 10.1371/journal.pone.0036842

**Published:** 2012-06-06

**Authors:** Katherine L. Ayres, Rebecca K. Booth, Jennifer A. Hempelmann, Kari L. Koski, Candice K. Emmons, Robin W. Baird, Kelley Balcomb-Bartok, M. Bradley Hanson, Michael J. Ford, Samuel K. Wasser

**Affiliations:** 1 Despartment of Biology, Center for Conservation Biology, University of Washington, Seattle, Washington, United States of America; 2 National Marine Fisheries Service, Northwest Fisheries Science Center, Seattle, Washington, United States of America; 3 Soundwatch Boater Education Program, The Whale Museum, Friday Harbor, Washington, United States of America; 4 Cascadia Research Collective, Olympia, Washington, United States of America; 5 Renton City Hall, Renton, Washington, United States of America; University of Western Ontario, Canada

## Abstract

Managing endangered species often involves evaluating the relative impacts of multiple anthropogenic and ecological pressures. This challenge is particularly formidable for cetaceans, which spend the majority of their time underwater. Noninvasive physiological approaches can be especially informative in this regard. We used a combination of fecal thyroid (T3) and glucocorticoid (GC) hormone measures to assess two threats influencing the endangered southern resident killer whales *(*SRKW*; Orcinus orca*) that frequent the inland waters of British Columbia, Canada and Washington, U.S.A. Glucocorticoids increase in response to nutritional and psychological stress, whereas thyroid hormone declines in response to nutritional stress but is unaffected by psychological stress. The inadequate prey hypothesis argues that the killer whales have become prey limited due to reductions of their dominant prey, Chinook salmon (*Oncorhynchus tshawytscha*). The vessel impact hypothesis argues that high numbers of vessels in close proximity to the whales cause disturbance via psychological stress and/or impaired foraging ability. The GC and T3 measures supported the inadequate prey hypothesis. In particular, GC concentrations were negatively correlated with short-term changes in prey availability. Whereas, T3 concentrations varied by date and year in a manner that corresponded with more long-term prey availability. Physiological correlations with prey overshadowed any impacts of vessels since GCs were lowest during the peak in vessel abundance, which also coincided with the peak in salmon availability. Our results suggest that identification and recovery of strategic salmon populations in the SRKW diet are important to effectively promote SRKW recovery.

## Introduction

Conservation management decisions often involve weighing the relative impacts of multiple, co-occurring anthropogenic and ecological pressures on wildlife health. Physiological measures provide valuable tools for evaluating the relative importance of such impacts [1], an essential first step to guide mitigation and evaluate its success.

The endangered population of southern resident killer whales *(Orcinus orca;* SRKW*)* that frequent the inland marine waters of southern British Columbia, Canada and Washington, U.S.A. (termed the Salish Sea) provide a case in point. The three southern resident “pods” each form long-term stable groups that frequent the Salish Sea for varying amounts of time from May through October [2]–[4]. From November through May, all three pods spend the majority of their time along the outer coast [3]. SRKWs are almost exclusively piscivorous, which distinguishes them from sympatric “transient” killer whales that forage on other marine mammals [5], [6]. A near 20% decline from 1995–2001 precipitated the SRKW being listed as an endangered population under the Canadian Species at Risk Act in 2001 [7] and the United States Endangered Species Act in 2005 [4], [8]. Both the US and Canadian SRKW Recovery Plans outline three main threats that may have contributed to the past decline and may currently slow recovery: vessel disturbance (“vessel impact hypothesis”), nutritional stress from inadequate prey availability (“inadequate prey hypothesis”), and exposure to persistent organic pollutants (“toxin hypothesis”) [9], [10]. Here we use noninvasive endocrine measures in SRKW scat to evaluate the inadequate prey and vessel impact hypotheses in an effort to help guide mitigation priorities.

The vessel impact hypothesis argues that exposure to a high abundance of vessel traffic is associated with behavioral changes, increased energy expenditure and/or foraging interference [11]–[16], resulting in psychological and/or nutritional stress. The SRKW are the focus of the whale watching industry in the inland waters of Washington and southern British Columbia, which includes a combination of private and commercial whale watching vessels. The whales are also exposed to private and commercial fishing boats, recreational powerboats, sailboats, kayaks, research vessels, military vessels and freight carrying ships. Reducing potential vessel impacts is complicated by the collective contribution of these vessels to U.S. and Canadian economies, along with treaty and international trade agreements. In 2011, NOAA Fisheries implemented federal regulations restricting the approach of vessels within 200 yards of killer whales in U.S. coastal waters (www.nwr.noaa.gov/Publications/FR-Notices/2011/upload/76FR20870.pdf) as well as prohibiting parking a vessel in the path of traveling killer whales.

The inadequate prey hypothesis argues that the SRKW population experiences times of prey limitation due to marked declines and fluctuations in the availability of their primary food source, adult Chinook salmon (*Oncorhynchus tshawytscha)* on the west coast of the United States and Canada [9], [10], [17], [18]. During the summer months, SRKWs eat a diet estimated to be 80–90% Chinook salmon [19], [20]. Adult Chinook salmon are the largest of the salmonids and have the highest caloric and fat content, which may explain the whales’ strong preference for them [19]. However, most Chinook salmon stocks in the eastern North Pacific are at a fraction of their historic levels due to a combination of historical overfishing, habitat loss and dams and other blockages to migration and large-scale climate variation [21], [22]. Long-term demographic studies show that SRKW survival [17], fecundity [18] and social cohesion [23], [24] are positively correlated with annual indices of Chinook salmon abundance. Salmon conservation and restoration is economically and politically complicated by a large number of factors that impact salmon throughout their complex life-cycle.

The toxin hypothesis stems from biopsy studies, revealing persistent organic pollutants in SRKW blubber that exceed an established health-effects threshold, presumably due to biomagnification in these long-lived, top-level predators [25]–[28]. Although the present study focuses on the inadequate prey and vessel impact hypotheses, impacts of these lipophilic toxicants on SRKW are likely tied to periods of food deprivation due to associated increases in fat metabolism [29], [30]. Eliminating legacy toxins in the international Salish Sea ecosystem is yet another economic, logistic and politically complicated task.

To test the inadequate prey and vessel impact hypotheses, we measured fecal glucocorticoid (GC) [31] and thyroid (triiodothyronine or “T3”) [32] hormone concentrations in relation to temporal changes in Chinook salmon availability and vessel traffic over a three-year period. The combination of GC and T3 hormone measures from the same sample are well suited to distinguish the relative contributions of psychological and nutritional stress to a population’s physiological health [33], [34]. GC concentrations rise in response to nutritional stress as well as a wide variety of psychological stressors, including circumstances triggering fight or flight or an animal’s perceived lack of control over its environment [34]–[37]. By contrast, T3 concentrations decrease in response to nutritional stress [38]–[40], but are largely unaffected by psychological stress [41]–[44].

Glucocorticoids are steroid hormones released from the adrenal cortex that help regulate a suite of physiological and behavioral coping mechanisms in response to nutritional as well as psychologically stressful situations [37], [45]. T3 is a modified amino acid that helps regulate metabolism [46]. In vertebrates, both hormones are excreted as metabolites in feces, at concentrations that reflect biological activity [31], [32]. However, the GC response to nutritional and other emergencies tends to be more rapid than the T3 response. Short-term nutritional emergencies cause a rise in GC concentrations that promote quick glucose mobilization followed by rapid metabolism and clearance of GCs from circulation once the stressor has passed. By contrast, sustained food deprivation causes a decrease in T3 concentrations, slowing metabolism to conserve energy stores [46].

**Figure 1:**
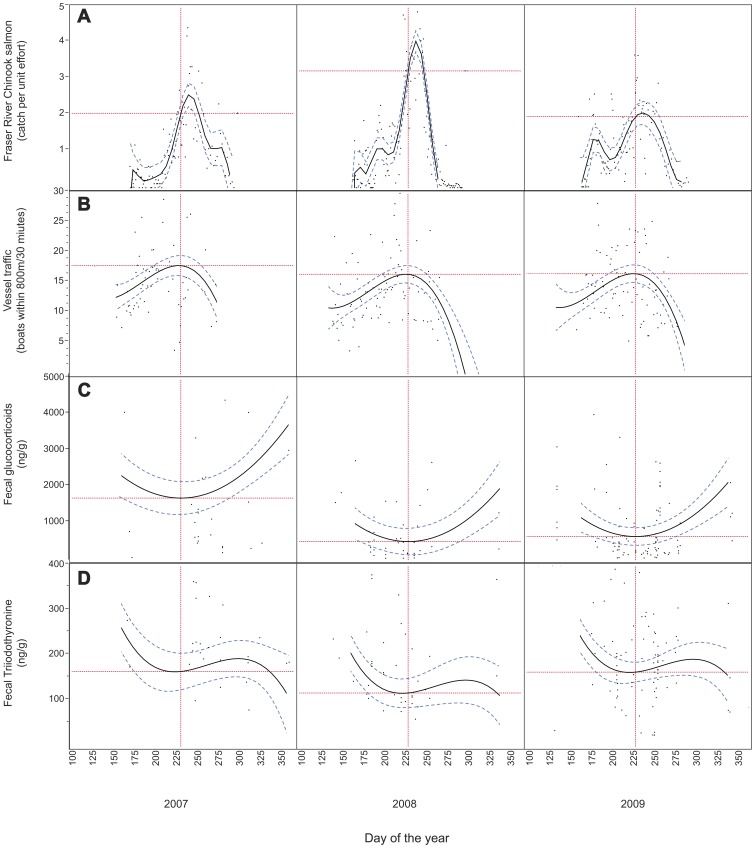
Temporal trends in variables used to test the inadequate prey and vessel impacts hypotheses. Temporal variation in Fraser River Chinook salmon catch per unit effort at the Albion test fishery (a); vessel traffic in proximity to Southern resident killer whales (b); physiological stress (indexed by fecal glucocorticoid concentrations) (c); nutrition (indexed by fecal triiodothyronine concentrations) (d). Trend lines determined using general linear model selection with predictor variables year, Julian date (linear, quadratic, cubic, etc.; see Table S1) and the interactions between year and Julian date parameters. Hashed lines indicate 95% confidence intervals. Dotted vertical lines indicate Julian day 230 (August 18), the time of maximum vessel traffic and approximately ten days before the maximum Chinook salmon catch each year. Horizontal dotted lines indicate dependent variable marginal means for each year on day 230 within the individual model.

While in the Salish Sea from May through September, the SRKW primarily eat Chinook salmon heading to the Fraser River system [20]. Fraser River Chinook salmon counts are relatively low when the whales first arrive sometime in the late spring and early summer, as are the number of vessels in the area (Figure 1a and 1b respectively). Both Fraser River Chinook salmon counts and vessel abundance peak around August-September, progressively declining thereafter. These coincident peaks allow us to use GC and T3 measures to distinguish between the inadequate prey and vessel impact hypotheses. Under the inadequate prey hypothesis, GC concentrations should be relatively high upon SRKW arrival when Fraser River Chinook salmon counts arelow. GC concentrations should reach their nadir around August-September–the peak of Fraser River Chinook salmon counts–and then increase as Fraser River Chinook salmon decline thereafter. The vessel impact hypothesis makes the opposite prediction. GC concentrations should be relatively low due to low vessel traffic when SRKW arrive in late Spring, peak around August-September with the peak in vessel abundance, and decline with declining vessel traffic thereafter. If prey availability and vessel impacts act cumulatively, we predict an interaction between Fraser River Chinook salmon counts and vessel abundance on GC concentrations. Specifically, GC concentrations should show a steeper positive correlation with vessel abundance during years of low Fraser River Chinook salmon returns.

The inadequate prey hypothesis also predicts a relation between T3 and Fraser River Chinook salmon; T3 should be positively correlated with Fraser River Chinook salmon. However, if the T3 response to nutrition is more protracted, we expect the T3 concentration of arriving whales to initially reflect the abundance and nutritional quality of the food source the whales were eating just prior to their arrival in the Salish Sea (e.g., during the previous 1–2 months). If the prior food source was relatively more nutritious, SRKW T3 concentrations at first arrival should still be high despite Fraser River Chinook salmon being relatively low at that time. Under those circumstances, we expect SRKW T3 concentrations to progressively decline from their time of first arrival, increase again around the peak in Fraser River Chinook salmon, and then decline continuously until the late fall departure. Since T3 is uncorrelated with psychological stress, T3 should only be correlated with vessel abundance if an increase in vessel abundance persistently interferes with killer whale foraging efficiency.

## Materials and Methods

### Ethics Statement

Fecal samples were collected in United States waters under National Marine Fisheries Service permits 532-1822-00, 532-1822 and 10045 and in Canadian waters under Marine Mammal License numbers 2008–16 and 2009–08 as well as Species at Risk Act permits numbered 91 and 102. Sample collection methods were approved by the University of Washington’s Institutional Animal Care and Use Committee (IACUC) although no permit was required, because the research was non-invasive.

### Study area and Population

The Salish Sea is an estuarine fjord habitat that supports a great diversity of species, including many salmon populations. The May through October SRKW occurrence in the Salish Sea coincides with annual Fraser River Chinook salmon migrations [47]–[49], which comprise 80–90% of the prey consumed by SRKWs during the summer [20]. The Fraser River system includes multiple rivers and tributaries throughout British Columbia that eventually converge and empty into the Strait of Georgia. Occasionally, SRKWs are also observed in coastal waters near the mouths of the Sacramento River and the Columbia River, two other U.S.A. west-coast river systems that currently support large Chinook salmon populations [8], [10]. However, these sightings most often occur during winter and early spring when sighting effort and diet data are both very limited.

The Center for Whale Research has maintained an annual photo-identification census of all the whales in the population, tracking age and life history stage for all individuals since the 1970s. The SRKW population is made up of three familial groups or pods: J, K and L. Each individual is identified by a unique combination of saddle patch and dorsal fin morphology and is designated alphanumerically with the letter representing its pod (J, K, or L) and the number its order of initial identification within the pod (e.g., J1; www.whaleresearch.com).

The three pods interact and interbreed with each other, but not with other killer whale populations [3], [50]–[52]. Each pod is made up of multiple matrilines–a highly stable group of individuals linked by maternal descent [3], [53], [54]. Neither males nor females disperse from their natal group [3], [53], [55]. Maternal pedigrees are well described through the annual census and many confirmed through a combination of mitochondrial and microsatellite DNA analyses [50], [52], [56]. Of the three pods, J pod spends the greatest amount of time in the Salish Sea.

In 2011, the SRKW population consisted of about 88 individuals (www.whaleresearch.com). In the 1960s and 1970s, approximately 50 SRKW individuals were live-captured from the population for marine aquaria [10], [57]–[59]. The population had recovered to pre-capture numbers by the early 1990s, but then experienced a near 20% decline from 1995–2001 that could not be explained by demographic effects from the live captures [60]. The decline resulted from increased mortality across all sex/age classes and several periods of low reproduction [4], [8].

### Sample Collections

Collection of floating fecal samples occurred from May through October in the Salish Sea: Haro, Rosario, Juan de Fuca, and Georgia Straits as well as Swanson Channel and Boundary Passage. Additional samples were opportunistically collected in November and December when some whales were in Puget Sound, Washington, U.S.A. Two research teams were involved in fecal collections. The first utilized a 6 m fiberglass motorboat (Boston Whaler) and a 6 m fiberglass motorboat with an open bow for detection dog sampling (Grady White). The second team utilized 6 m and 7 m rigid-hulled inflatable boats with a bow platform (Avon and Zodiac respectively).

Samples were located using two different sampling methods: focal animal follows and detection dog assisted sampling of one or more clustered individuals. The first research team conducted focal animal follows in 2007 and for two months in 2008. Detection dog techniques were then implemented for one month in 2008 and all of 2009. The second research team conducted focal animal follows exclusively. Focal animal follows were conducted by following closely behind the whale, searching for scat floating in the fluke prints–a series of calm circles of displaced water left after a whale surfaces and then submerges [19], [20]. We confirmed the target whale’s identity whenever possible using published photo-identification catalogs [61], [62].

Detection dog sampling was conducted using a modification of previously published methods [63] for fecal sample collection in marine environments [64]. Use of a detection dog enabled us to sample at an average distance of 400 meters from the target whale(s), minimizing any potential disturbance from the research vessel. The detection dog was selected for its obsessive drive to play with a ball. Sample localization was paired with a brief (<2 min) play reward with a ball. Once the dog associated sample localization with receipt of the reward, it would change its behavior to an alert searching mode as soon as the target scent was detected. Training the dog on samples from a variety of individuals taught the dog to generalize its alert response to scent common to all individuals of the target species [63].

During sampling, the dog rode on the bow of the vessel with the dog handler. The driver maneuvered the vessel in transects perpendicular to the wind and downwind from a group of whales or the area that they previously swam through. When the vessel was in the cone of the scent emanating downwind of the floating scat, the dog indicated sample detection by changing his behavior from a relaxed sit or stand to leaning over the bow of the vessel with tensed muscles, anticipating a reward. The dog maintained this position as long as the scent concentration increased from low to high. The dog alerted the handler as soon as the scent concentration began to change from high to low concentration by standing erect and turning in the direction of the more concentrated scent. The handler communicated this to the driver, who made an en course correction confirmed by the dog’s return to a tensed muscle position on the bow. As we got close to the scat, the dog often stood up and began to whimper, presumably because the scent was surrounding the vessel and he could no longer follow a concentration gradient. Throughout this whole process, crewmembers visually scanned for the sample floating on the water’s surface.

Fecal samples were identified via appearance and odor. Killer whale feces are observed as clumped patches, having a mucousy and/or semi-cohesive texture. Killer whale feces are usually brown or green, but can also appear grey, yellow or orange. Samples often have a characteristic fishy odor that can be recognized with experience. We have observed SRKW fecal samples floating on the water’s surface for up to 45 minutes. If scat is defecated below the surface or the surface tension is disturbed than the fecal pieces sink. Once a sample was identified, it was collected with a scoop or fine mesh net mounted on a telescoping pole. The scoop proved optimal for samples floating on the surface, because it minimized sample disturbance and provided better sample recovery compared to the net (Ayres unpublished data). Nets were more effective for collecting samples below the surface. When samples were collected with scoops, excess water was carefully poured off. The sample was then transferred to a 50 ml polypropylene screw-top vial, promptly centrifuged at 1,000 rpm for approximately 5 minutes and the excess water decanted from the fecal pellet. When collected with a net, the water was drained off and the sample transferred to the 50 ml polypropylene screw-top vial. Approximately 2–5 sub-samples for separate DNA analyses were taken whenever possible with sterile cotton swabs or small pieces of sterile gauze. All samples were stored on ice for up to 12 hours while in the field, and then at −20°C upon return to the field station. Sub-samples were shipped on dry ice at the end of each field season to the Center for Conservation Biology for hormone analyses and to NOAA, Northwest Fisheries Science Center for DNA analyses. All samples were stored until extraction at −20°C.

### Hormone Extraction and Radioimmunoassay

In the lab, each sample was thawed once and centrifuged at 2,200 rpm for 20 minutes. Excess salt-water was decanted from the fecal pellet, taking care not to lose the fecal pellet. The samples were then lyophilized for 48 hours in a Labconco FreeZone Freeze Dry System. Samples were lyophilized prior to extraction and hormone concentrations expressed per gram dry weight to control for inter-sample variation due to diet and variable moisture [65]. Freeze-dried fecal material was thoroughly mixed and up to 0.1 g weighed and transferred to a new 50 ml polypropylene screw-top tube for extraction. Samples smaller than 0.02 g dried weight were excluded from analysis to avoid inflation effects of low sample mass on hormone concentrations [66], [67]. Fecal material was extracted in 15 ml of 70% ethanol according to previously published methods [32], with one modification. The fecal pellet was only extracted once since previous validation showed very low hormone concentrations in the second extract for GCs and T3 in killer whale samples (Ayres unpublished data). The extract was then stored at −20°C until hormone analysis.

Radioimmunoassay was performed to measure fecal hormone metabolites using ^125^I corticosterone RIA kits (#07-120103; MP Biomedicals, Costa Mesa, CA) and MP Biomedicals’ Total T3 coated tube assay kits (#06-B254216) for GC metabolites and T3, respectively. The T3 assay was previously validated for killer whales [32]. The GC assay [31] was validated for killer whales in the present study (see below). Commercial controls from each assay kit were used to assess inter-assay coefficients of variation. Commercial T3 controls were prepared as previously described [32].

### Hormone Assay Validations

Standard parallelism and accuracy tests [68] were performed on a pooled extract from 5 different killer whale fecal samples. Parallelism tests compare the slope of a curve generated from serially diluted fecal extracts to that of the standard curve; parallel slopes indicate that hormone metabolites are being reliably measured across their range of concentration. Accuracy tests plot concentrations of standards spiked with fecal extract against those of unspiked standards. A slope of 1.0, after adjusting for the added hormone concentration in the added extract, indicates that products in the extract are not interfering with antibody binding in the radioimmunoassay.

Challenge experiments are also used in validation studies to assess whether excreted hormone metabolites reflect biological activity. A tropic hormone (e.g., adrenocorticotropic hormone for GC or thyroid stimulating hormone for T3) is injected to induce secretion of the respective target hormone, which should then be measured as a significant increase in excretion of its metabolites in feces. We were unable to obtain permission to conduct such challenge studies on captive killer whales. So, we used the alternative of obtaining an opportunistic sample from a severely emaciated, physiologically stressed adult male killer whale that stranded on the coast of Kauai, Hawai’i, expecting its GC concentration to be markedly elevated compared to that of adult males in the SRKW population. A similar opportunistic challenge was not possible for thyroid hormone because we could not ascertain the degree to which disease contributed to the whale’s emaciation.

### DNA Analyses

DNA analyses were conducted on all fecal samples to confirm species, sex and individual identification at the Northwest Fisheries Science Center, NOAA, in Seattle, Washington, USA. DNA extraction and analyses were performed according to previously published methods [52]. Species was confirmed by fragment length of 16 s ribosomal DNA. Sex was confirmed by amplification of the SRY and ZFX genes [69]. Individual identification was made by amplification of 26 polymorphic microsatellite loci, subsequently matched to other fecal and biopsy samples acquired from known individual killer whales [52]. If a genotype could not be matched to a known individual, the genotype was recorded and given a unique identification number, therefore, that unknown individual could still be included in the analyses to control for pseudoreplication. Occasionally, unique genotypes could also be assigned to pod, if there was only one pod in the area at the time of sampling. Thus, using genotypes we were able to track samples that were from the same individual and sometimes identify the genotype to pod even if the identity of the individual could not yet be determined.

### Prey and Vessel Traffic Measures

Approximately 80–90% of the SRKWs diet from May through September is made up of Fraser River Chinook salmon [20]. Therefore, we compared changes in hormone concentrations over time with changes in the Department of Fisheries and Oceans’ Fraser River Albion test fishery, which is the most consistent data set available to index relative availability of Fraser River Chinook salmon [70]. Data are reported as catch per unit effort (CPUE). Chinook salmon CPUEs on days when the test fishery did not operate were estimated by averaging the CPUE from the day prior and the day after.

Vessel abundance was quantified using data collected by The Whale Museum’s Soundwatch Boater Education Program. Observers count the total number of vessels observed within a half mile (ca. 800 m) of any whale in view, at 30-minute intervals during day light hours, with the aid of laser range finders to measure distances [71]. Vessel data were gathered from May through September in 2007 and 2008 and through October in 2009. There is approximately a 24-hour lag time between hormone secretion in blood and its excretion in feces in large mammals [31], [32], [72], making the previous days’ vessel counts most relevant to hormone concentrations in a given sample. Therefore, vessel traffic was averaged throughout a given day and compared to hormone concentrations from the following day.

### Distinguishing between Inadequate Prey and Vessel Impacts

To test the inadequate prey and the vessel impact hypotheses as well as their potential interaction, we used general linear mixed effects models to test the effects of year, sex, pod, Fraser River Chinook salmon counts and vessel abundance as main effects, and all two-way interactions of main effects on natural log transformed fecal GC and T3 concentrations. Individual differences were controlled in these analyses by including individual identity as a random effect in the models. We also tested GC concentrations as a predictor variable for T3 and vice versa to test for inter-hormone effects.

All statistical analyses were conducted using the model fit application in the JMP 9 statistical package. Candidate models were compared using the R^2^ adjusted of the model [73], where the best-fit model was indicated by the highest R^2^ adjusted value.

The Albion test fishery is approximately 140 km travel distance from the west side of San Juan Island, the whales’ primary feeding area where the majority of our samples were collected. We used a best-fit model to estimate the time lag from the date of SRKW fecal collection until the date the Chinook salmon were caught at the test fishery. As a cross-check, the best fit time lag was compared to travel time for a fish to swim from prime whale foraging grounds off the west side of San Juan Island [20] to the Albion test fishery based on documented Chinook salmon swim speeds multiplied by the distance traveled [74]. Both analyses indicated a 10-day time lag, and this was the lag we subsequently used in our analyses predicting hormone levels.

### Addressing Pseudoreplication

On twelve occasions, multiple samples were collected from the same individual on the same day. For these twelve cases, hormone concentrations were averaged between the samples or the largest, more representative sample was used for that individual on that day.

## Results

### Sampling

We collected 154 fecal samples that were large enough (>0.02 g) to be confidently assayed for hormone concentrations (see Methods). Of these, 138 samples were successfully genotyped for sex determination. Twice as many males as females were sampled in 2007, while males and females were sampled in roughly equal proportions in 2008 and 2009 (Table 1). Of the 154 samples, 113 were identified to pod. J pod, which is the most frequently occurring pod in the Salish Sea, was sampled most often (Table 1), followed by K and then L pod. Pods were sampled in similar proportions in 2008 and 2009.

**Table 1:** Distribution and percent of fecal samples successfully identified to sex and pod.

	Sex (percent identified)		Pod (percent identified)		
Year	Male	Female	J Pod	K Pod	L Pod
2007	18 (67%)	9 (33%)	13 (46%)	3 (10%)	12 (42%)
2008	17 (50%)	17 (50%)	17 (50%)	7 (21%)	10 (29%)
2009	37 (48%)	40 (52%)	25 (49%)	11 (22%)	15 (29%)

### Validations

Both corticosterone and T3 assays exhibited excellent parallelism; slopes of serially diluted extracts were not significantly different from the slopes of the standard curves (GC: F_1,7_ *= *0.41 p = 0.54; T3: F_1,9_ = 2.89, p  =  0.12). Fifty percent binding of the radioactively labeled hormone occurred at target dilutions of 1∶240 for GC and 1∶30 for T3 concentrations. Both T3 and corticosterone assays exhibited good accuracy at their target dilutions (Linear regression; GC: slope  = 1.2, R^2^ = 0.98; T3: slope  = 1.09 and R^2^ = 1.0), indicating that substances in fecal extract do not interfere with hormone binding. Inter-assay coefficients of variation for T3 and GCs were 14.6% and 10%, respectively. Intra-assay coefficients of variation for T3 and GCs were 1.9% and 3%, respectively.

The opportunistic hormone challenge study showed the stranded male killer whale in Hawai’i had a fecal GC concentration that was 27 times higher than the average male SRKW (Figure S1). This result suggests that fecal GC concentration is a reliable index of biological activity.

### Distinguishing between Inadequate Prey and Vessel Impacts

Figure 1 summarizes the annual and seasonal patterns of Fraser River Chinook salmon CPUE, vessel traffic, fecal GC and fecal T3 patterns from 2007 to 2009. Each variable was examined separately to assess how it changed within and between years during the study period. Raw data are presented in Figure 1 with trend lines determined using general linear model selection based on maximum likelihood model comparisons. Each variable was analyzed as a response to year, Julian date and higher orders of Julian date (quadratic, cubic, etc.) along with their interactions (Table S1). Fraser River Chinook CPUE was best fit by a 9^th^ order polynomial of Julian date across years (Figure 1a). A 9th order polynomial was necessary to capture the timings of multiple runs of different Chinook subpopulations returning to the Fraser River through the Albion test fishery [70]. Fraser River Chinook CPUE varied markedly between years. Early season (June) Chinook CPUE was lowest in 2007, intermediate in 2008 and highest in 2009 (Figure 1a). Peaks in Chinook runs (ca. Julian date 240 or August 28th) were intermediate in 2007, highest in 2008, and lowest in 2009. However, the width of the August peak was also narrowest in 2008, followed by 2007, and broadest in 2009.

Mean vessel abundance in proximity to whales did not differ significantly between years. The best-fit model for explaining vessel patterns was a 3^rd^ order polynomial of Julian date (Figure 1b). On any given day from June through September, average vessel traffic was consistently between 10–18 boats around groups of whales (Figure 1b). Vessel traffic progressively increased to its peak around Julian day 230 (August 18^th^) and then steadily declined into the fall.

To explore temporal patterns in physiological stress, the entire GC data set was used to test linear models of GCs as a response to year and Julian date (Table S1). Fecal GCs (which index the combination of acute psychological and nutritional stress) over time was best predicted by a 2^nd^ order quadratic of Julian date (Figure 1c). GC concentrations were always intermediate when the whales arrived in the spring, and Fraser River Chinook were relatively low. GC concentrations progressively declined thereafter until Julian date 230 (August 18^th^)–approximately 10 days before the annual peak in Fraser River Chinook CPUE. GC concentrations increased from that point as salmon declined into the fall and winter. The highest observed GC concentrations occurred in November and December (Figure 1c). Average annual GC concentrations were comparable across years after controlling for Julian date.

**Figure 2:**
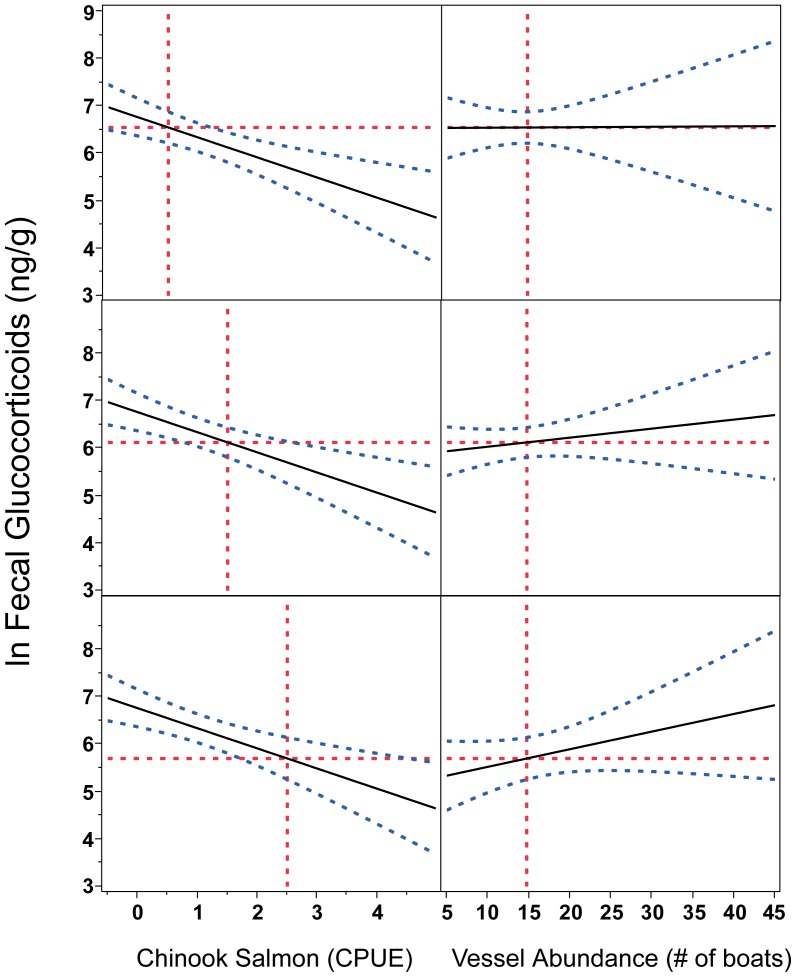
Physiological stress correlates with year, Chinook availability, vessel abundance and an interaction between Chinook and vessel abundance. According to the best-fit mixed effects model, glucocorticoid concentrations decreased with increased Chinook salmon CPUE, after taking into account a 10-day lag time for fish to swim from the study site to the test fishery (column A). The best-fit model also includes an interaction between Chinook counts and vessel abundance on glucocorticoids, whereby fecal glucocortiods are always high at times of low Chinook counts. However, an increase in glucocorticoids with increasing vessel abundance is observed only during times of relatively high Chinook counts (column B set to the Chinook value indicated by the vertical line in the corresponding panel of column A). The y-axis represents glucocorticoid concentration marginal means predicted from the best-fit model. The hashed blue lines indicate 95% confidence intervals. Vertical red dotted lines indicate Julian day 230 (August 18), the time of maximum vessel traffic and approximately ten days before the maximum Chinook salmon catch each year. Horizontal red dotted lines indicate dependent variable marginal means for each year on day 230 within the model.

We tested for effects of prey and vessel traffic on GC concentrations by fitting fecal GC concentrations to Fraser River Chinook CPUE, vessel abundance, Julian date, sex, pod and fecal T3 concentrations, including individual identity as a random effect. The best-fit models are presented in Table 2, however more detailed model selection data can be found in Tables S2 and S3. Fecal GC concentrations were best modeled as a response to year, Fraser River Chinook CPUE (with a 10-day time lag), vessel abundance in proximity to whales and the interaction of prey and vessel abundance (Table 2; GC Top model A). Chinook CPUE was the only significant main effect in this model, however less variance was explained by the model if any of the other parameters were removed. There was a highly significant negative relationship between GC concentrations and Fraser River Chinook CPUE each year; GC concentrations consistently decreased as Chinook counts increased (Figure 2a). Both year and Chinook CPUE were significant if vessel abundance and its interactions were removed, with GCs being significantly lower in 2007 compared to 2009 (Table 2; GC Top model B). Sex, pod and fecal T3 concentrations did not improve any of the tested models.

**Table 2:** Best-fit general linear mixed effects models explaining southern resident killer whale fecal glucocorticoid (GC) and triiodothyronine (T3) concentrations.

Model	Response	n	Parameter	Estimate	SE	p	R^2^Adj
GC	ln(GCs)	81	Year [2007]	0.31	0.43	0.39	0.75
top model A			Year [2008]	0.11	0.35	0.65	
			Chinook salmon (10-day lag)	−0.42	0.12	<0.001*	
			Vessel abundance	0.02	0.02	0.44	
			Chinook x Vessels	0.02	0.02	0.29	
			Individual (Random)				
							
GC	ln(GCs)	81	Year[2007]	0.63	0.29	0.04*	0.71
top model B			Year[2008]	−0.05	0.22	0.84	
			Chinook salmon(10-day lag)	−0.37	0.11	<0.01*	
			Individual (Random)				
							
							
T3	ln(T3)	79	Sex [Female]	25.62	11.58	<0.03*	0.51
top model A			Year [2007]	41.65	27.19	0.13	
			Year [2008]	−59.40	20.80	<0.01*	
			Julian date	−1.26	0.29	<0.0001*	
			Individual (Random)				

Similar to GCs, temporal patterns in the entire T3 data set were examined in response to year and various orders of Julian date (Table S1). Fecal T3 (presumed to index long-term nutritional status) was best fit by a third order quadratic of Julian date across years (Figure 1d). T3 concentrations were consistently highest (indicating relatively good nutrition) in the spring when the whales arrived in the Salish Sea (Figure 1d). T3 concentrations progressively declined from time of arrival until Julian date 230 (August 18^th^) followed by a slight but sustained upturn that began coincident with the Fraser River Chinook salmon peak (Figure 1a), but never rose to the spring arrival levels within any given year. T3 concentrations then progressively declined into the late fall/early winter. SRKW arrived with the highest T3 concentrations in 2007, but also showed the greatest percent decline over the entire study season in that year. Mean T3 was lowest in 2008 compared to 2007 and 2009. Although 2008 had the highest peak in Chinook CPUE, the 2008 peak was also the narrowest (Figure 1a).

**Figure 3:**
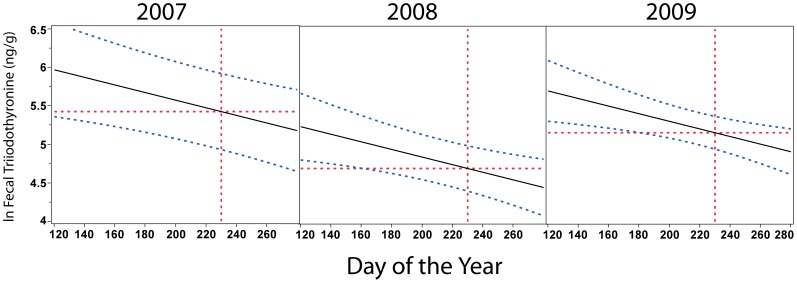
Nutrition correlates with sex, year and Julian date. Nutritional status, indexed by fecal triiodothyronine concentrations, is highest when the southern resident killer whales return to the Salish Sea in the spring and declines throughout the summer into the fall and winter. The y-axis represents triiodothyronine concentration marginal means predicted from the best-fit mixed effects model after controlling for individual and sex. The hashed blue lines indicate 95% confidence intervals. Vertical red dotted lines indicate Julian day 230 (August 18), the time of maximum vessel traffic and ten days before maximal Fraser River Chinook salmon catch each year. Horizontal red dotted lines indicate dependent variable marginal means for each year on day 230 within the model.

To test for effects of prey and vessel traffic on T3 concentrations, the T3 data set was restricted to the tested predictor variables: Fraser River Chinook CPUE, vessel abundance, Julian date, sex, pod and fecal GC concentrations, with individual ID included as a random effect. Fecal T3 concentrations were best modeled as an additive response to sex, year and Julian date (Table 2). Females had significantly higher average T3 concentrations in the model, as St. Aubin et al. [75] also reported for bottlenose dolphins. The best-fit model showed a linear response of T3 to Julian date, after controlling for sex. T3 concentrations were highest when the whales arrived in the spring with a steady decline into fall (Figure 3). Overall, T3 marginal means were highest in 2007, intermediate in 2009 and lowest in 2008 for any given day of the year (horizontal lines in Figure 3). Fraser River Chinook CPUE, vessel abundance, pod and fecal GC concentrations did not improve any of the tested models.

## Discussion

The temporal pattern in GC concentrations closely corresponds to relative Fraser River Chinook salmon counts from the time SRKW first arrive in the Salish Sea. This pattern appeared to result from a rapid GC responsiveness to prey availability. GC concentrations reached their nadir when Fraser River Chinook salmon (lagged by 10 days) peaked at the test fishery even though vessel abundance was also peaking around this time. GC concentrations then progressively rose to their highest levels of the year as Fraser River Chinook salmon declined, even though vessel numbers in proximity to the whales also markedly declined. When prey and vessel abundance indices were tested directly, GCs were significantly correlated with Fraser River Chinook salmon. Vessel abundance and its interaction with prey improved model performance as indicated by the amount of variance explained, however neither vessel abundance or the interaction were significant as parameters within the model. When vessel abundance and its interaction was removed from the model, year became significant, with 2007–the year with the lowest average salmon counts–being lower than 2009. This suggests that some combination of year, salmon availability and vessel abundance may be required to fully explain variance in GCs, however our sample size may have had insufficient statistical power to demonstrate that.

In contrast to GCs, T3 concentrations were not directly correlated with Fraser River Chinook counts, but were instead associated with Julian date. Temporal patterns in T3 concentrations across years indicate that SRKW nutrition is consistently highest when the whales first arrive in the Salish Sea during spring/early summer. Since Fraser River salmon counts were relatively low at that time, this suggests that the SRKW may consistently be foraging on an early spring, nutrient-rich food source just prior to their late spring arrival in the Salish Sea. T3 concentrations progressively declined from the time of SRKW arrival until the whales’ late fall/early winter departure. When the entire data set was used and did not include sex as a parameter, there was a slight increase in T3 starting in August, roughly coincident with the peak in Fraser River Chinook salmon, but T3 concentrations never reached those observed when the whales first arrived in the Salish Sea. The estimated T3 decline with Julian date was linear when the data were restricted by sex, potentially due to the restricted sample size that limited the statistical power to show a higher order relationship.

The temporal trend in T3 concentrations within and between years suggest that the sampled SRKWs might be feeding on a nutritious early spring food source acquired prior to their arrival in the Salish Sea. The trend further suggests that the whales become somewhat food limited during the course of the summer. This result is somewhat unexpected, because the more confined waterways of the Salish Sea, combined with large runs of salmon returning through the area would seem to provide easier foraging opportunities for the whales than the outer coast. Nonetheless, the declining trend in T3 levels at least suggests the possibility that the early spring period when the whales are typically in coastal waters might be a more important foraging time than was previously believed.

The spring range of SRKW is not well defined but available information indicates that their range includes the coastal waters of California, Oregon, Washington and British Columbia [8, NMFS unpublished data]. Several stocks of Chinook occur in these coastal waters in the spring [76]. Some of the most abundant Chinook stocks available to the whales in the spring are the Columbia River spring Chinook [77], and if the whales are foraging on these stocks, that may contribute to the elevated spring T3 concentrations prior to the whales’ arrival in the Salish Sea. These early spring Chinook are “interior race” salmon known to have particularly high fat content to sustain their long spawning migrations upstream to interior river systems [78], [79]. In contrast to the summer period, direct observation of the coastal feeding events are very limited. However, the available information does suggest that the whales may be feeding on Columbia River salmon. In particular, the only scale samples collected from foraging killer whales off the Washington coast in March were interior Columbia River Chinook salmon (n = 2; Hanson unpublished data). In addition, some SRKWs have been observed foraging near the mouth of the Columbia in late March, when the spring run Chinook salmon stocks return to the Columbia River [80]. Our results therefore reinforce the importance of gaining a better understanding of the whale’s diet during this potentially important time period, and suggest the possibility that these spring-run stocks might be of particular importance for the nutrition of this population.

The end of 2007 through 2008 appeared to represent the poorest overall nutritional state of the SRKW population during our three-year study. The whales left the Salish Sea in 2007 following the most precipitous T3 decline and GC elevation over the three years (Figure 1c and 1d). Their T3 concentrations upon arrival in late spring 2008 were the lowest observed during that time of year over the three-year study period and remained low throughout 2008. This period also corresponded with the highest number of deaths and lowest number of births and surviving calves observed during our three-year study. Eight whales went missing from December of 2007 through October 2008, two of which were reproductive age females (Center for Whale Research unpublished data) and included a visually emaciated pregnant female (L67; Ayres et al. in preparation). Loss of multiple reproductive age females is uncommon in long-lived mammals and is particularly detrimental to population recovery in a population of this size. It is also noteworthy that while the Fraser River Chinook salmon peak in 2008 had the highest amplitude of the three study years, the peak was relatively brief (Figure 1a). Perhaps this brief pulse in relative fish availability during 2008 overwhelmed the predator, actually making a relatively small proportion of the total fish returns accessible to the whales that year. Consistent Chinook availability throughout the season, as occurred in 2009 (Fig. 1a), may be much more important to SRKW sustained nutrition compared to high numbers of fish that are only available for a short period of time.

Oritz et al. [81] found that captive bottlenose dolphins responded to a 38 hour fast by elevating lipid metabolism to spare lean tissue. They observed an initial decline in serum T3 followed by recovery, although the trend was not significant. There was, however, an increase in biologically inactive reverseT3 (rT3) by 38 hours, suggesting that such conversion to rT3 may protect dolphins from excess cellular metabolism during caloric restriction. Our results suggest that more sustained periods of reduced food availability in SRKW likely results in a lowering of basal T3, which is probably a critical strategy for conserving energy and slowing the need for lipid metabolism. Such a strategy may be crucial during sustained periods of food decline, given the importance of lipids as a long-term energy store in addition to their importance in buoyancy and thermoregulation.

Despite previous reported pod differences in movement patterns and the locations of prey consumed in the winter and early spring [28], including pod as a predictor variable did not improve any of the models we tested. While these preliminary analyses do not indicate a significant difference in physiological trends between pods, J pod was represented more often in our data than K and L pods, suggesting that more data may be needed to address pod differences in physiology.

Our findings that glucocorticoids are correlated with an index of Chinook salmon availability are consistent with studies indicating a high percentage of Chinook salmon in the SRKW diet [19], [20], [82] as well as correlations of SRKW demographic trends with coast-wide indices of Chinook abundance [17], [18]. Our results suggest that prey availability has a greater physiological impact on SRKWs than does vessel traffic. However, we cannot yet rule out a cumulative effect of vessel traffic on the overall SRKW stress response, particularly during years of relatively low Fraser River Chinook abundance. Exposure to toxicants may also add to these cumulative effects if food deprivation promotes metabolism of lipid stores, releasing sequestered toxicants into circulation. Combined, these results suggest that promoting salmon recovery is vital to the long-term persistence of SRKW. Conservation of early spring salmon runs consumed by SRKW prior to arrival in the Salish Sea may be especially important to these recovery efforts. Future studies should aim to better identify these early spring food sources to better target recovery efforts.

It is a modern reality that anthropogenic impacts and ecology are forever intertwined. As anthropogenic disturbances continue to affect wildlife, it is important for conservation biologists and managers to prioritize mitigation efforts. To this end, conservation biologists need tools that better clarify anthropogenic and ecological impacts on the health of endangered populations before devastating demographic incidents occur. This study shows that combining GC and T3 hormone measures enables investigators to partition the relative impacts of psychological and nutritional stressors, along with their short versus long-term metabolic consequences. As such, these combined tools offer more timely evaluation of anthropogenic disturbances, their ecological significance and provide means to monitor the success of mitigation efforts in free-ranging vertebrates.

### Disclaimer

The findings and conclusions in this article are those of the author(s) and do not necessarily represent the views of the National Oceanic and Atmospheric Administration.

## Supporting Information

Figure S1 **Biological relevance of fecal glucocorticoids in a stranded killer whale.** All genetically confirmed male Southern resident killer whale fecal glucocorticoid concentrations (n = 36) were compared to a killer whale that stranded in Hawai’i. The killer whale was severely emaciated and later euthanized. The stranded killer whale had exceptionally high fecal glucocorticoid concentrations (ca. 28 times higher than the male SRKW average), indicating stress-induced adrenal activation. Similar results from a right whale tangled in a fishing net were observed by Hunt et al (2006).
(TIFF)

Table S1 **Best-fit general linear models testing annual and seasonal patterns in Fraser River Chinook salmon, vessel traffic, fecal glucocorticoid and triiodothyronine concentrations.**
(DOC)

Table S2 **Model comparisons for the final set of mixed effects models tested to explain fecal glucocorticoid (GC) concentrations.**
(DOC)

Table S3 **Model comparisons for the final set of mixed effects models tested to explain fecal triiodothyronine (T3) concentrations.**
(DOC)
