# Supplementary figures and images for: Distinguishing the Impacts of Inadequate Prey and Vessel Traffic on an Endangered Killer Whale (Orcinus orca) Population
Source: PLoS One. 2012 Jun 6;7(6):e36842. doi: 10.1371/journal.pone.0036842 (PMC3368900; doi:10.1371/journal.pone.0036842)

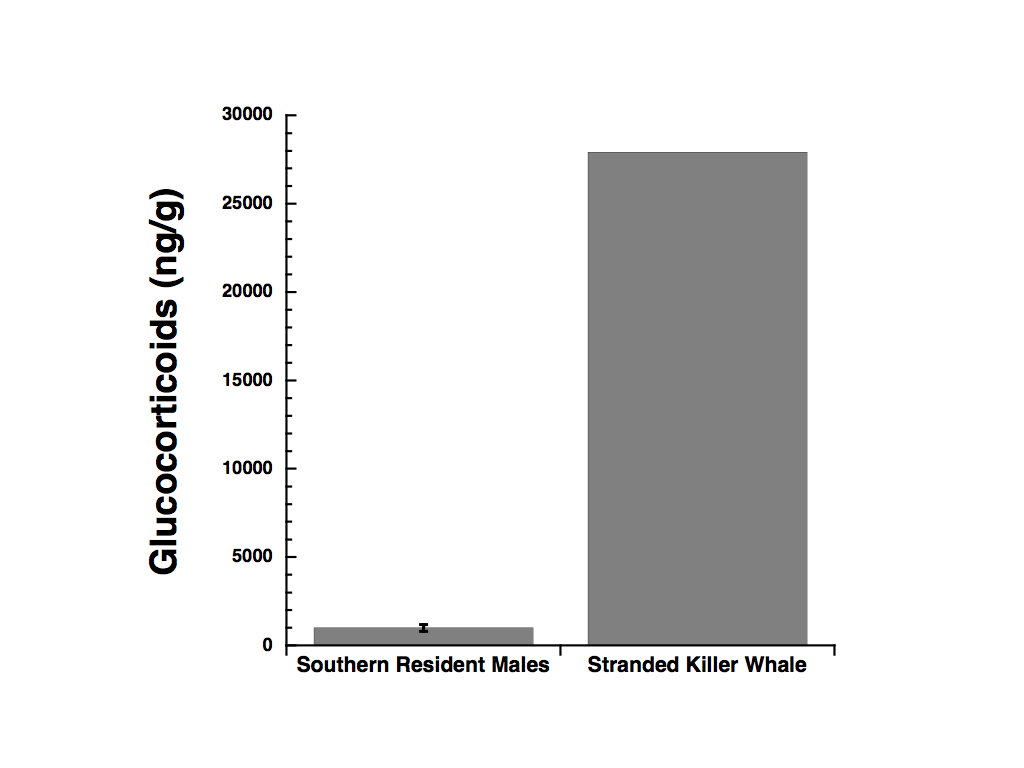

Supplement: Figure S1 — Biological relevance of fecal glucocorticoids in a stranded killer whale. All genetically confirmed male Southern resident killer whale fecal glucocorticoid concentrations (n = 36) were compared to a killer whale that stranded in Hawai’i. The killer whale was severely emaciated and later euthanized. The stranded killer whale had exceptionally high fecal glucocorticoid concentrations (ca. 28 times higher than the male SRKW average), indicating stress-induced adrenal activation. Similar results from a right whale tangled in a fishing net were observed by Hunt et al (2006). (TIFF) [file pone.0036842.s001.tif]
