# Supplementary material for: Distinguishing the Impacts of Inadequate Prey and Vessel Traffic on an Endangered Killer Whale (Orcinus orca) Population
Source: PLoS One. 2012 Jun 6;7(6):e36842. doi: 10.1371/journal.pone.0036842 (PMC3368900; doi:10.1371/journal.pone.0036842)
Supplement: Table S1 — Best-fit general linear models testing annual and seasonal patterns in Fraser River Chinook salmon, vessel traffic, fecal glucocorticoid and triiodothyronine concentrations. (DOC) [file pone.0036842.s002.doc]

Table S1. Best-fit general linear models testing annual and seasonal patterns in Fraser River Chinook salmon, vessel traffic, fecal glucocorticoid and triiodothyronine concentrations.

| Model | Response | Unit | n | Parameters | Delta AICc* |
| --- | --- | --- | --- | --- | --- |
| Chinook top model | Fraser River Chinook | CPUE | 332 | year + Julian date (9^th^ order polynomial) + year:date interactions | 0 |
| Vessel traffic top model A | Vessel traffic | vessels w/n 800m/30 minutes | 241 | Julian date + Julian date^2^ + Julian date^3^ | 0 |
| Vessel traffic top model B | Vessel traffic | vessels w/n 800m/30 minutes | 241 | year + Julian date + Julian date^2^ + Julian date^3^ | +1.99 |
| GCs top model | Glucocorticoids | ng/g | 162 | year + Julian date + Julian date^2^ | 0 |
| T3 top model A | Triiodothyronine | ng/g | 161 | Julian date + Julian date^2^ + Julian date^3^ | 0 |
| T3 top model B | Triiodothyronine | ng/g | 161 | year + Julian date + Julian date^2^ + Julian date^3^ | +1.23 |

*Delta AICc is compared to the best-fit model set to “0” for each set of models.
