# Supplementary material for: Distinguishing the Impacts of Inadequate Prey and Vessel Traffic on an Endangered Killer Whale (Orcinus orca) Population
Source: PLoS One. 2012 Jun 6;7(6):e36842. doi: 10.1371/journal.pone.0036842 (PMC3368900; doi:10.1371/journal.pone.0036842)
Supplement: Table S2 — Model comparisons for the final set of mixed effects models tested to explain fecal glucocorticoid (GC) concentrations. (DOC) [file pone.0036842.s003.doc]

Table S2. Model comparisons for the final set of mixed effects models tested to explain fecal fecal glucocorticoid (GC) concentrations.

| Glucocorticoid Models^1^ (n = 81) | R^2^adj |
| --- | --- |
| *Individual* (Random) | 0.54 |
| *Individual* + year | 0.51 |
| *Individual* + year* + Fraser River Chinook (10-day lag)* | 0.71 |
| *Individual* + year + Fraser River Chinook (10-day lag) + vessel abundance | 0.73 |
| *Individual* + year + Fraser River Chinook (10-day lag)* + vessel abundance + (Chinook x vessels) | 0.75 |

^*^Parameter found significant at alpha = 0.05 within the model.
