# Supplementary material for: Distinguishing the Impacts of Inadequate Prey and Vessel Traffic on an Endangered Killer Whale (Orcinus orca) Population
Source: PLoS One. 2012 Jun 6;7(6):e36842. doi: 10.1371/journal.pone.0036842 (PMC3368900; doi:10.1371/journal.pone.0036842)
Supplement: Table S3 — Model comparisons for the final set of mixed effects models tested to explain fecal triiodothyronine (T3) concentrations. (DOC) [file pone.0036842.s004.doc]

Table S3. Model comparisons for the final set of mixed effects models tested to explain fecal triiodothyronine (T3) concentrations.

| Triiodothyronine Models^2^ (n = 79) | R^2^adj |
| --- | --- |
| *Individual* (Random) | < 0 |
| *Individual* (Random) + sex | < 0 |
| *Individual* (Random) + sex* + year* | 0.22 |
| *Individual* (Random) + sex* + year*+ Julian date* | 0.63 |

^*^Parameter found significant at alpha = 0.05 within the model.
